# Associação entre Hipertensão Arterial Sistêmica com Marcadores Laboratoriais, Composição Corporal, Apneia Obstrutiva do Sono e Variabilidade da Frequência Cardíaca em Adultos Obesos

**DOI:** 10.36660/abc.20220728

**Published:** 2023-06-23

**Authors:** Clarcson Plácido Conceição Santos, Laura Souza Lagares, Sarah Rafaela Mascarenhas Santos, Mariana Sousa de Pina Silva, Rodrigo Colares de Macedo, Luiz Alberto Bastos de Almeida, Eric Simas Bomfim

**Affiliations:** 1 Escola Bahiana de Medicina e Saúde Pública Grupo de Pesquisa em Doenças Metabólicas, Exercício Físico e Tecnologias em Saúde Salvador BA Brasil Escola Bahiana de Medicina e Saúde Pública – Grupo de Pesquisa em Doenças Metabólicas, Exercício Físico e Tecnologias em Saúde, Salvador, BA – Brasil; 2 Universidade Estadual de Feira de Santana Departamento de Educação Física Feira de Santana BA Brasil Universidade Estadual de Feira de Santana – Departamento de Educação Física, Feira de Santana, BA – Brasil

**Keywords:** Hipertensão, Biomarcadores, Composição Corporal, Apneia Obstrutiva, Obesidade, Frequência Cardíaca, Adultos

## Abstract

**Fundamento:**

A hipertensão arterial sistêmica (HAS) é uma doença multifatorial, altamente prevalente e associada a riscos à saúde.

**Objetivo:**

O objetivo deste estudo foi investigar a associação entre HAS e marcadores laboratoriais, antropométricos, de variabilidade da frequência cardíaca (VFC) e de apneia obstrutiva do sono e, em segundo plano, analisar a sensibilidade e especificidade das variáveis que são fatores independentes na associação.

**Métodos:**

Estudo transversal com 95 pacientes obesos atendidos em um ambulatório de referência em obesidade em Salvador, BA, Brasil. Os dados da HAS foram obtidos dos prontuários eletrônicos. A amostra foi estratificada em Grupo Normotenso (GN) e Grupo Hipertenso (GH), sendo medidos marcadores laboratoriais, composição corporal, polissonografia e VFC para avaliar a associação da HAS com as variáveis preditoras. Para as análises, adotou-se p<0,05.

**Resultados:**

A média da idade do GN foi de 36,3 ± 10,1 e GH 40,4 ± 10,6 anos, 73,7% eram mulheres no GN e 57,9% no GH; 82,4% no GH apresentavam resistência à insulina. No modelo de regressão logística multivariado com ajustes para idade, sexo, altura e saturação de oxi-hemoglobina, a HAS foi inversamente associada à glicose plasmática em jejum mg/dL (odds ratio [OR] = 0,96; intervalo de confiança de 95% [IC] = 0,92-0,99) e área de gordura visceral (AGV) cm^2^ (OR = 0,98; IC 95% = 0,97-0,99). A área sob a curva AGV foi de 0,728; IC 95% (0,620-0,836) e glicemia de jejum 0,693; IC 95% (0,582-0,804).

**Conclusão:**

Menores concentrações de AGV e glicemia de jejum foram inversamente associadas à HAS. Além disso, tanto a glicemia de jejum quanto o AGV mostraram alta sensibilidade para triagem de HAS.

**Figura Central:**
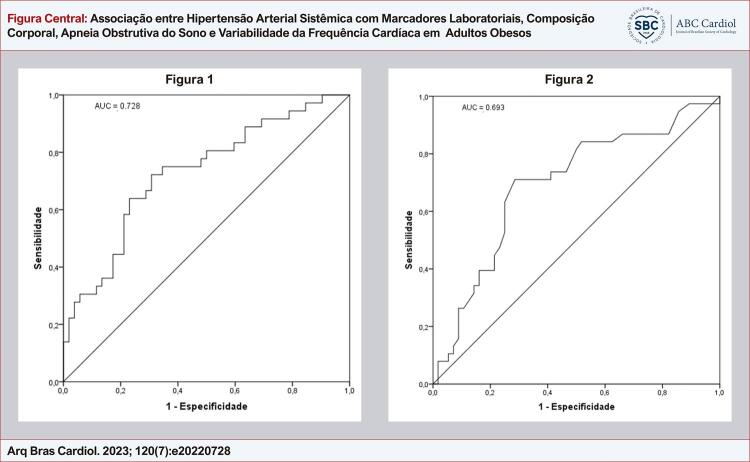
: Associação entre Hipertensão Arterial Sistêmica com Marcadores Laboratoriais, Composição Corporal, Apneia Obstrutiva do Sono e Variabilidade da Frequência Cardíaca em Adultos Obesos

As figuras apresentam os dados referentes à sensibilidade e especificidade da hipertensão arterial sistêmica com a gordura visceral e glicemia de jejum, respectivamente, além de seus pontos de corte para triagem de hipertensão arterial sistêmica.

## Introdução

A hipertensão arterial sistêmica (HAS) é uma doença multifatorial, podendo ser proveniente de fatores ambientais e/ou genéticos, como a falta de atividade física, obesidade e hábitos alimentares.^1^ De acordo com a Organização Mundial da Saúde, estima-se que 1.28 bilhão de adultos com idades entre 30 e 79 anos em todo o mundo tenham hipertensão.^2^ Atualmente a HAS elevada está associada a um maior risco de mortalidade e é um fator significativo para intercorrências de eventos renais e cardiovasculares.^3^

A hipertensão pode ser induzida por possíveis alterações advindas da obesidade, como a estimulação de mecanismos que contribuem para este estado hipertensivo, podendo ser alterações a níveis hormonais, inflamatórios e endoteliais.^4^ A obesidade está associada a uma diminuição na expectativa de vida e sua prevalência tem se tornado um grande problema de saúde mundial, visto que o ganho de peso excessivo predispõe um aumento do risco de várias doenças, dentre essas, as doenças cardiovasculares, cerebrovasculares e metabólicas todas associadas a HAS.^5 , 6^

Dados prévios da literatura descrevem que alguns fatores devem ser considerados preditores de riscos para a ocorrência da HAS, entre eles estão a síndrome da apneia obstrutiva do sono, índice de massa corporal (IMC), circunferência de cintura (CC), área de gordura visceral abdominal, variabilidade da frequência cardíaca,^7 - 9^ alguns marcadores bioquímicos laboratoriais e comorbidades associadas.^10 , 11^ Em virtude da quantidade de variáveis e suas possíveis associações, ou não, torna-se necessário uma pesquisa mais aprofundada sobre as relações entre esses dados e a HAS, de forma que seja possível obter preditores mais confiáveis e independentes para tomadas de decisão na prática clínica, facilitando o prognostico de HAS nessa população.

Distúrbios respiratórios do sono como a apneia obstrutiva podem acelerar a progressão dos níveis de pressão arterial presente em adultos, especialmente de forma aguda podendo ser devido a hipóxia durante a noite.^7^ Já os mecanismos provocados por um maior valor de IMC, CC, massa de gordura corporal e glicemia podem provocar a estimulação do sistema nervoso simpático, alterações no sistema renina-angiotensina-aldosterona, aumento de marcadores inflamatórios e outros fatores responsáveis pelo equilíbrio no sistema circulatório, dessa forma, podendo associar-se a HAS.^12 - 15^ A hipertensão também está relacionada com a desregulação autonômica e, visto que a VFC pode ser caracterizada também por uma maior ativação simpática pode-se dizer que este seria o mecanismo associado a HAS.^16^

Diante do exposto, o objetivo do presente estudo foi investigar as associações da HAS com marcadores bioquímicos laboratoriais, medidas antropométricas e de composição corporal, variabilidade da frequência cardíaca e apneia obstrutiva do sono em adultos obesos e, em um segundo plano, de analisar a sensitividade e a especificidade das variáveis que são fatores independentes na associação, bem como seus respectivos pontos de corte.

## Materiais e métodos

### Desenho do estudo e amostra

Este estudo foi baseado em dados de corte transversal de 95 pacientes com idade ≥ 21 anos com diagnostico de obesidade e eletivos à cirurgia bariátrica em uma clínica particular de cirurgia e tratamento da obesidade na cidade de Salvador no Brasil. Os dados foram coletados no período de maio de 2016 a agosto de 2018. Pacientes com déficit cognitivo e sem a totalidade dos dados clínicos e laboratoriais não foram incluídos no estudo. Os voluntários do estudo foram categorizados em dois grupos de acordo com o diagnóstico clínico de hipertensão arterial sistêmica: grupo de normotensos (GN) e grupos de hipertensos (GH). O estudo foi submetido e aprovado pelo Comitê de Ética em Pesquisa da Escola Bahiana de Medicina e Saúde Pública/EBMSP, parecer número 1.530.178. Os autores declaram que todos os experimentos foram conduzidos seguindo a Declaração de Helsinque.

### Instrumentos de medidas

#### Composição Corporal

Os dados de composição corporal foram mensurados por bioimpedância elétrica octopolar através do equipamento InBody 720 (inBody Canada Corp, Ottawa, Ontario, Canada) cumprindo os procedimentos especificados na literatura. Essa bioimpedância utiliza oito eletrodos, dois em contato com a palma (E1, E3) e o polegar (E2, E4) de cada mão e dois em contato com a parte anterior (E5, E7) e posterior (E6, E8) da planta de cada pé. Cinco impedâncias segmentares (braço direito, braço esquerdo, perna direita, perna esquerda e tronco) são medidas a 1, 5, 50, 250, 500 e 1000 KHz. Os pontos de contato do corpo com os eletrodos foram previamente limpos com um tecido eletrolítico recomendado pelo fabricante e os participantes orientados a cumprirem as seguintes normas de preparação: estar em jejum de pelo menos 4 horas, não consumir álcool 48h antes do teste, não realizar exercício de intensidade moderada à elevada nas 12 horas antes da avaliação, estar em um estado de hidratação adequada antes de realização da avaliação, não utilizar peças metálicas ou implantes dentários com metal (quando passíveis de serem removidos) e não ingerir café. Como resultado da bioimpedância, as seguintes variáveis foram determinadas: massa corporal total (kg), massa de gordura corporal (kg), massa musculo esquelético (kg), área de gordura visceral (cm^2^) e índice de massa corporal (kg/m^2^). Idade (anos), estatura (cm), circunferência da cintura (cm) e circunferência do quadril (cm) foram coletadas da base de prontuários do sistema da clínica.

#### Variáveis bioquímicas

Os marcadores bioquímicos coletados foram HOMA-IR, insulina, glicose em jejum, colesterol total, colesterol HDL e triglicérides. O colesterol total, HDL, e triglicérides foram quantificados no soro, por meio de sistema colorimétrico. Os valores de referência foram considerados os da metodologia aplicada pelo laboratório, que são baseadas nos valores apresentados pela Sociedade Brasileira de Diabetes e Sociedade Brasileira de Cardiologia.^17^ Todas os dados foram coletados da base de prontuários do sistema da clínica, no pré-operatório.

#### Análise da variabilidade da frequência cardíaca

Para os registros dos batimentos cardíacos foi utilizado o cardiofrequencimetro (v800 *heart rate monitor* da Polar®), calculado através da razão entre o intervalo RR, transferidos para um programa de computador com o objetivo de analisar a VFC através do *software* polar *precision performance* que foram importados para o Kubios *software* HRV (versão 2.0), utilizado para calcular os métodos lineares do domínio de tempo e frequência. Para a análise da VFC no domínio do tempo foi utilizado a raiz quadrada da média das diferenças ao quadrado entre os intervalos RR normais (RMSSD) e o desvio padrão da média de todos os intervalos RR normais (SDNN). Para a análise da VFC no domínio da frequência foram utilizados os componentes espectrais de baixa frequência (LF, 0,04-015 Hz) e alta frequência (HF, 0,15 a 0,40 Hz) em unidades normais (LFun e HFun, respectivamente), o que representa um valor em relação a cada componente espectral em relação à potência total menos os componentes muito baixa frequência (VLF), e a relação entre esses componentes (razão LF/HF).

A análise espectral foi calculada utilizando o algoritmo da transformada rápida de Fourier. Os participantes da amostra foram convidados a permanecerem em repouso, na posição de decúbito dorsal, sem exposição a luz excessiva e em um ambiente sem ruídos por 10 minutos para análise de um ponto de corte de 5 minutos, verificando no período pré-operatório.

#### Apneia obstrutiva do sono

Os dados de polissonografia foram obtidos através de equipamento computadorizado da *Respironics* (Sistema *Healthdyne Alice* 4), sendo o laudo revisto, independentemente, por especialistas treinados. Um terceiro especialista foi consultado no caso de inconsistências no laudo final. O exame foi conduzido durante toda a noite, em sono espontâneo, sem nenhuma sedação ou privação do sono. Foram registrados: eletroencefalograma (eletrodos C3, C4), oculograma (O1, O2), eletromiograma (eletrodos nas regiões mentoniana, submentoniana e MMII), eletrocardiograma, fluxo aéreo (termistor nasal e bucal), esforço respiratório (cinta torácica e abdominal), ronco (microfone no queixo) e posição do corpo (sensor na cinta torácica).^18^

A saturação da oxi-hemoglobina foi mensurada através da oximetria de pulso. Os eventos respiratórios foram assim definidos: apneia, como a interrupção do fluxo aéreo por 10 segundos ou mais, e hipopneia, como a redução de 50% ou mais do fluxo aéreo inspiratório por período ≥ 10 segundos, associado a um decréscimo superior a 3% na saturação da oxi-hemoglobina e/ou a um micro despertar.

As apneias mistas foram incluídas no Índice de Apneia e Hipopneia (IAH), e definidas como aquelas que apresentavam ausência de esforço respiratório no início do período, seguido de aumento gradual. O IAH foi obtido através de exame polissonográfico, dividindo o total de eventos respiratórios pelas horas de sono. Os pacientes foram classificados de acordo com o IAH em: sem apneia – menos de 5.0 eventos/hora de sono; com apneia leve – entre 5.0 e 14.9 eventos/hora de sono; com apneia moderada – entre 15.0 e 30.0 eventos por/hora de sono e com apneia grave – mais de 30.0 eventos/hora de sono.

#### Plano estatístico

Análises descritiva e analítica foram realizadas através do software *Statistical Package for Social Sciences program, version 14.0 for Windows* (SPSS Inc, Chicago, IL). Comparações entre pacientes normotensos e com hipertensão arterial sistêmica foram conduzidas baseado no diagnóstico clínico. A normalidade das variáveis foi verificada através da estatística descritiva e do teste de Kolmogorov-Smirnov. As variáveis categóricas foram expressas em valores absolutos e porcentagens e o teste de qui-quadrado usado para testar as diferenças entre as variáveis qualitativas. Variáveis contínuas com distribuição normal foram expressas como média e desvio padrão e as com distribuição não normal como mediana e intervalo interquartil. O test-t para amostras independentes ou o teste Mann-Whitney U foram utilizados para testar as diferenças entre as variáveis quantitativas. Modelos multivariados de regressão logística foram usados para estimativa da associação entre a hipertensão arterial sistêmica com composição corporal e marcadores laboratoriais. Para elaboração dos modelos de ajustes, as variáveis que apresentaram p<0,2 foram consideradas. A odds ratios foram ajustadas para idade, sexo, estatura e saturação de oxi-hemoglobina. *Receiver Operating Characteristic Curve* ( *ROC curve* ) foram usadas para estimativa da sensibilidade e especificidade entre hipertensão arterial sistêmica e área de gordura visceral abdominal e glicemia plasmática em jejum, assim como, os seus respectivos pontos de corte. Para inferência estatística, um valor de p<0,05 foi adotado.

## Resultados

Um total de 95 participantes de ambos os sexos foram selecionados para o estudo. O GN foi composto por 57 participantes (60%), e o GH de 38 participantes (40%), com uma média de idade 36,3 ± 10,1 e 40,4 ± 10,6 anos, respectivamente (p = 0,062). A tabela 1 apresenta as características dos pacientes de acordo com o diagnóstico clínico de HAS, categorizados como GN e GH. Comparados com os pacientes do GN, os pacientes do GH apresentaram maior massa corporal, IMC, CC, massa de gordura corporal (MGC) e AGV. A porcentagem de pacientes diagnosticados com resistência à insulina foi maior no GH. Os grupos foram homogêneos em relação aos dados laboratoriais, polissonografia, gravidade da apneia obstrutiva do sono (SAOS) e parâmetros da VFC.

**Tabela 1:** – Características dos pacientes de acordo com o diagnóstico de hipertensão arterial sistêmica

	GN (n = 57)	GH (n = 38)	Valor de p
Idade (anos)	36,3 (10,1)	40,4 (10,6)	0,062
**Gênero n (%)**			
Homem	15 (26,3)	16 (42,1)	0,123
Mulher	42 (73,7)	22 (57,9)	
**Composição corporal**			
Massa corporal (kg)	113,3 (18,5)	124,6 (25)	**0,013**
Altura (cm)	166,8 (8,3)	169,6 (8,4)	0,114
IMC (kg/m^2^)	40,5 (4,6)	42,9 (6)	**0,027**
CC (cm)	117,3 (12,1)	124,6 (18,3)	**0,024**
MME (kg)	32,7 (7,4)	35,8 (7,6)	0,058
MGC (kg)	55,1 (9,2)	60,7 (14,9)	**0,027**
AGV (cm^2^)	202,8 (54,1)	262,2 (78,6)	**0,0001**
**Dados Laboratoriais**			
Colesterol total (mg/dl)	197 (57)	201 (36,7)	0,657
HDL (mg/dl)	49,5 (11,8)	48,3 (11,5)	0,636
Triglicerídeos (mg/dl)	146 (74)	175,7 (113,4)	0,162
Glicemia em jejum (mg/dl)	96,9 (35,4)	105,5 (26,2)	0,180
Homa-IR	5 (3,9)	6,4 (3,9)	0,135
**Comorbidades n (%)**			
Diabetes Mellitus	5 (8,8)	6 (15,8)	0,338
Resistência Insulínica	30 (57,7)	28 (82,4)	0,020
SAOS	34 (65,4)	24 (72,7)	0,633
**Medidas de Polissonografia**			
IAH (eventos/h)	8,4 [3,8 – 15,7]	9,9 [4 – 17,5]	0,389
Frequência de SAOS	19 [13,7 – 27,5]	18 [12 – 29,2]	0,754
SO (%)	95 [93 – 96]	94 [92 – 95,7]	0,174
**Severidade da SAOS n (%)**			
< 5 eventos/h	18 (34,6)	9 (27,3)	0,513
5 – 30 eventos/h	31 (59,6)	20 60,6)	
> 30 eventos/h	3 (5,8)	4 (12,1)	
**VFC Parameters**			
**Domínio de tempo**			
RR Médio	760,4 [638–849]	726 [647–814]	0,660
SDNN (ms)	70,7 [37,7–274]	84 [43,7–422]	0,522
RMSSD (ms)	64 [21,6–340]	117 [33,8–550]	0,373
pNN50 (ms)	18,4 [1,8–50]	13,6 [3,4–40,4]	0,861
**Domínio de Frequência**			
LF (ms^2^)	56 [41–75]	51 [27–84,8]	0,601
HF (ms^2^)	43,6 [23,6–56]	46 [15,2–64,8]	0,443
LF/HF	1,3 [0,74–3,8]	1,4 [0,54–8,5]	0,898

IMC: índica de massa corporal; CC: circunferência abdominal; MME: massa muscular esquelética; MGC: massa de gordura corporal; AGV: área de gordura visceral; HDL: lipoproteínas de alta densidade; SAOS: apneia obstrutiva do sono; IAH: índice de apneia e hipopneia; SO: saturação de oxi-hemoglobina; VFC: variabilidade da frequência cardíaca; SDNN: desvio padrão da média de todos os intervalos RR normais; RMSSD: raiz quadrada da média das diferenças ao quadrado entre os intervalos RR normais; LF: baixa frequência; HF: alta frequência. O teste qui-quadrado foi utilizado para analisar

A tabela 2 apresenta associações significantes (p<0,05) da HAS com medidas de composição corporal, dados laboratoriais e de comorbidades, através de análises multivariadas não ajustadas e ajustadas. As variáveis massa corporal, massa muscular esquelética (MME), IMC, CC, triglicérides, HOMA-IR e resistência à insulina não apresentaram diferenças estatísticas na análise de regressão logística multivariada. No modelo de análise final, após ajustes para covariáveis, incluindo idade, sexo, estatura e saturação de oxiemoglobina, a associação entre HAS e composição corporal foi (OR = 0,98, 95% confidence interval (CI) = 0,97-0,99) para área de gordura visceral e para marcadores laboratoriais (OR = 0,96, 95% CI = 0,92-0,99) para glicemia plasmática em jejum. Ambas as variáveis demonstraram serem as únicas independentemente associadas com HAS.

**Tabela 2:** – Modelo de regressão logística multivariada das variáveis de gordura corporal, laboratoriais e comorbidade entre pacientes obesos com e sem hipertensão arterial sistêmica

Variáveis	Modelo Inicial			Modelo Final*		
	
	β	OR (95% IC)	p	β	OR (95% IC)	p
Massa Corporal	-0,089	0,91 (0,75 – 1,10)	0,362	-	-	-
AGV	-0,015	0,98 (0,97 – 0,99)	**0,011**	-0,014	0,98 (0,97 – 0,99)	0,026
MME	0,154	1,16 (0,84 – 1,61)	0,356	-	-	-
IMC	-0,026	0,97 (0,76 – 1,25)	0,842	-	-	-
CC	0,005	1,00 (0,94 – 1,06)	0,882	-	-	-
Triglicerídeos	-0,001	0,99 (0,99 – 1,00)	0,684	-	-	-
Glicemia em jejum	-0,035	0,96 (0,93 – 0,99)	**0,043**	-0,040	0,96 (0,92 – 0,99)	0,047
Homa - IR	0,017	1,07 (0,86 – 1,19)	0,836	-	-	-
Resistência Insulínica	-1,167	0,31 (0,08 – 1,21)	0,093	-	-	-

*O modelo final inclui idade, sexo, altura e saturação de oxi-hemoglobina. AGV: área de gordura visceral; MME: massa muscular esquelética; IMC: índice de massa corporal; CC: circunferência abdominal.

**Figura 1:**
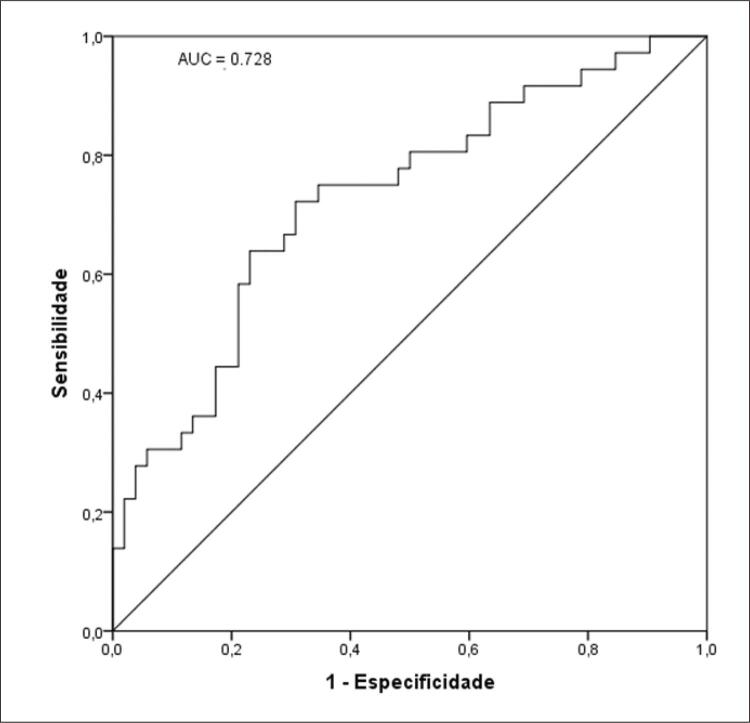
– Curva ROC para área de gordura visceral como triagem para HAS. Área soba curva (AUC) = 0,728; IC 95% (0,620 – 0,836). Ponto de corte para HAS: > 220,3 cm2.

As figuras 1 e 2 apresentam os dados relacionados com a sensibilidade e especificidade da HAS com a área de gordura visceral e a glicemia plasmática em jejum, respectivamente, além de seus pontos de corte para *screening* da HAS. A área de gordura visceral apresentou área sob a curva = 0,728 (95% CI = 0,62-0,84) e ponto de corte para HAS: > 220,3 cm^2^, enquanto a glicemia plasmática em jejum apresentou área sob a curva = 0,69 (95% CI = 0,58-0,80) e ponto de corte para HAS: > 95 mg/dl.

**Figura 2:**
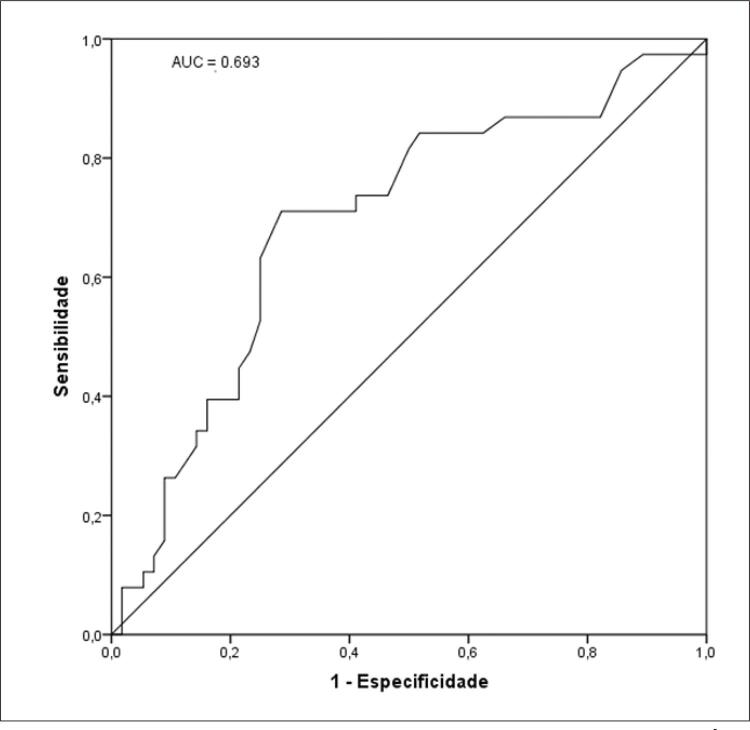
– Curva ROC para triagem de glicemia de jejum para HAS. Área sob a curva (AUC) = 0,693; IC 95% (0,582 – 0,804). Ponto de corte para HAS: > 95 mg/dl.

## Discussão

No presente estudo, análises comparativas entre os grupos demonstraram que medidas de composição corporal, dados laboratoriais e de comorbidade foram maiores no GH. No entanto, as únicas variáveis independentemente associadas a HAS foi a área de gordura visceral e a glicemia plasmática em jejum. As forças dessas associações descritas nas análises não ajustadas foram levemente alteradas após ajustes para potenciais variáveis de confusão. Estes resultados fornecem suporte adicional acerca da importância da manutenção dos baixos níveis de armazenamento de gordura visceral abdominal, assim como, do controle da glicemia plasmática em jejum como potenciais fatores de proteção para a HAS.

Medidas de composição corporal, marcadores bioquímicos e comorbidades avaliados nesse estudo, impactam de diferentes formas nos mecanismos relacionados com a HAS. Estudos prévios corroboram com os nossos achados, nesse sentido, Chandra et al.,^19^ demonstraram que medidas maiores de IMC foram significativamente associadas a HAS incidente nos participantes.^19^ Nesse sentido, Lee et al.,^20^ encontraram que a cada aumento de 1kg/m^2^ no IMC houve também um aumento de 19% no risco de hipertensão arterial e Holmes et al.,^21^ mostraram que para cada aumento em 1kg/m^2^ no IMC a pressão arterial sistólica aumentou em 0,70mmHg.^21^ Uma provável explicação para a associação da medida de IMC com a HAS, sabendo que trata-se de um índice com pontos de corte para a classificação de obesidade com uma boa acurácia na predição,^22^ é o fato de que o fenótipo obeso, mesmo que metabolicamente saudável, está diretamente ligado à um risco aumentado para a hipertensão,^23 , 24^ visto que há mecanismos patológicos como hiperinsulinemia, estimulação do sistema nervoso simpático e níveis anormais de adipocitocinas que afetam o endotélio vascular, responsável pela manutenção da homeostase vascular.^12^

Ainda tratando de medidas de composição corporal, em nossos achados a circunferência de cintura também demonstrou associação com a HAS, assim como no estudo de Guilherme at al.,^25^ o qual demonstrou que em adolescentes brasileiros a CC obteve uma associação positiva como indicador antropométrico independente para HAS, sendo que, aqueles classificados com obesidade central tiveram 130% mais chances de ter a pressão arterial elevada quando comparados com os adolescentes sem o diagnóstico de obesidade abdominal. Carba et al.,^26^ encontraram que a cada aumento de 1cm na CC as chances de hipertensão aumentaram em 5% para mulheres sem sobrepeso e 3% para mulheres com sobrepeso.^26^ Sendo a CC um indicador de obesidade abdominal,^27^ pode-se dizer que uma possível explicação para a associação da CC com a HAS esteja relacionada com o excesso de depósitos de gordura nessa região, visto que o tecido adiposo visceral tem um importante papel na ativação do sistema renina-angiotensina-aldosterona, o que pode influenciar na hemodinâmica central e sistêmica.^13^

Como podemos observar, a mudança de um fenótipo normotenso para hipertenso envolve diferentes fatores. Além das variáveis já citadas, a MGC também interfere na hemodinâmica de forma que a distribuição de gordura pode ditar o risco de doenças cardiovasculares.^28^ Dessa maneira, Han et al.,^29^ encontraram que em comparação com indivíduos normotensos, o percentual de gordura corporal foi significativamente maior no grupo de hipertensos.^29^ Park et al.,^30^ também demonstraram que indivíduos com alta porcentagem de gordura corporal foram associados ao aumento do risco de hipertensão mesmo com baixo IMC, CC ou relação cintura-quadril, sendo que o aumento do risco foi proporcional ao aumento do percentual.^30^ Nesse caso, através do aumento da MGC os níveis no plasma de biomarcadores inflamatórios como proteína C-reativa e interleucinas também podem aumentar, o que consequentemente, pode predispor o desenvolvimento de doenças cardiovasculares, incluindo a hipertensão.^14^

Como mencionado anteriormente, a distribuição de gordura pode ditar o risco de doenças cardiovasculares e, ainda nesse sentido, indivíduos com maior tecido adiposo visceral e depósitos de gordura ectópica tem uma prevalência ainda maior de distúrbios metabólicos como a hipertensão.^31 , 32^ Nas figuras 1 e 2 pode-se visualizar a área sobre a curva ROC para sensibilidade e especificidade para área de gordura visceral e glicemia plasmática em jejum encontradas no nosso estudo, as quais demonstram que ambas as variáveis obtiveram associações independentes com a HAS, destacando principalmente, a área de gordura visceral. O tecido adiposo visceral em excesso realiza a secreção de hormônios e moléculas que acentuam doenças cardiovasculares e também se torna resistente à insulina e leptina, podendo contribuir para resistência vascular e disfunção do sistema nervoso simpático.^31^ Já o tecido adiposo intra-abdominal em níveis elevados pode ser considerado como parte de fenótipo cujo resultado associa-se a uma alteração disfuncional do tecido adiposo subcutâneo e armazenamento ectópico de triglicerídeo, levando essa alteração morfológica a fazer parte de um conjunto de fatores de risco cardiometabólicos.^33^

Sabendo que a resistência à insulina pode contribuir para a resistência vascular e disfunção do sistema nervoso simpático,^31^ é importante destacar sua relação com os níveis de glicemia plasmática em jejum, visto que, à medida que a glicemia em jejum aumenta, o índice de sensibilidade a insulina diminui.^34^ Em um estudo realizado em japoneses foi observado que altos níveis de glicose em jejum foi independentemente e significativamente associado à hipertensão, sendo que a taxa de risco em participantes com glicemia acima ou igual a 7,0 mmol/l foi de 1,79 comparado com os participantes com glicemia menor que 5,6 mmol/l.^15^ Esses resultados corroboram com os nossos achados, já que a glicemia plasmática em jejum foi associada independentemente a HAS.

As associações de glicemia plasmática em jejum e gordura visceral abdominal com a HAS descritos no presente estudo têm implicações práticas potenciais para intervenções de tratamento para melhorar os resultados em pacientes com obesidade e são provavelmente generalizáveis para populações em todo o mundo. No entanto, existem limitações para determinar se as associações estatísticas são causais e a direção das associações deve ser levada em consideração antes de se chegar a conclusões definitivas. Como o estudo é observacional, não é possível descartar os efeitos de confusão residual ou não mensurada como explicação para os resultados. Além disso, o desenho transversal não permite determinar se o quadro clínico de HAS precedeu ou foi influenciado pelo perfil metabólico e morfológico. É possível que as associações observadas da HAS com os marcadores bioquímicos e de composição corporal sejam bidirecionais.

## Conclusão

Em conclusão, o presente estudo demonstra uma associação inversa e independente entre a concentração de glicose plasmática em jejum e a área de gordura visceral abdominal com HAS em pacientes obesos. Além disso, tanto a glicemia de jejum quanto a área de gordura visceral apresentaram alta sensibilidade para triagem de HAS. Os resultados chamam a atenção para a importância de intervenções para melhorar o controle de variáveis bioquímicas e de composição corporal, prevenir alterações na glicemia plasmática e atenuar o aumento da gordura visceral abdominal em pacientes com obesidade.
