# Supplementary material for: Associação entre Hipertensão Arterial Sistêmica com Marcadores Laboratoriais, Composição Corporal, Apneia Obstrutiva do Sono e Variabilidade da Frequência Cardíaca em Adultos Obesos
Source: Arq Bras Cardiol. 2023 Jun 23;120(7):e20220728. [Article in Portuguese] doi: 10.36660/abc.20220728 (PMC10365017; doi:10.36660/abc.20220728)
Supplement: Translation

# Translation: Association between Arterial Hypertension and Laboratory Markers, Body Composition, Obstructive Sleep Apnea and Autonomic Parameters in Obese Patients

**Authors:** Clarcson Plácido Conceição Santos, Laura Souza Lagares, Sarah Rafaela Mascarenhas Santos, Mariana Sousa de Pina Silva, Rodrigo Colares de Macedo, Luiz Alberto Bastos de Almeida, Eric Simas Bomfim

**Affiliations:** 1 Brazil Escola Bahiana de Medicina e Saúde Pública – Grupo de Pesquisa em Doenças Metabólicas, Exercício Físico e Tecnologias em Saúde, Salvador, BA – Brazil; 2 Brazil Universidade Estadual de Feira de Santana – Departamento de Educação Física, Feira de Santana, BA – Brazil

**Keywords:** Hypertension, Biomarkers, Body Composition, Sleep Apnea, Obstructive, Obesity, Heart Rate, Adults

## Abstract

**Background:**

Systemic arterial hypertension (SAH) is a multifactorial disease, highly prevalent and associated with health risks.

**Objective:**

The purpose of this study was to investigate the association between SAH and laboratory, anthropometric, heart rate variability (HRV), and obstructive sleep apnea markers and, secondarily, to analyze the sensitivity and specificity of the variables that are independent factors in the association.

**Methods:**

Cross-sectional study with 95 obese patients treated at an obesity referral clinic in Salvador, BA, Brazil. SAH data were obtained from electronic medical records. The sample was stratified in the Normotensive Group (NG) and the Hypertensive Group (HG), and laboratory markers, body composition, polysomnography, and HRV were measured to evaluate the association of SAH with the predictor variables. For the analysis, p<0.05 was adopted.

**Results:**

The average age of the NG was 36.3 ± 10.1 and HG 40.4 ± 10.6 years; 73.7% were women in the NG and 57.9% in HG; 82.4% in HG had insulin resistance. In the multivarious logistics regression model with adjustments in age, sex, height, and oxyhemoglobin saturation, SAH was inversely associated with fasting plasma glucose mg/dL (odds ratio [OR] = 0.96; 95% confidence interval [CI] = 0.92-0.99) and visceral fat area (VFA) cm^2^(OR = 0.98; 95% CI = 0.97-0.99). The area under the VFA curve was 0.728; CI 95% (0.620-0.836); fasting plasma glucose 0.693;CI 95% (0.582-0.804).

**Conclusions:**

Lower VFA and fasting plasma glucose concentrations were inversely associated with SAH. In addition, fasting plasma glucose and VFA showed a high sensitivity for SAH screening.

**Central Illustration:** : Association between Arterial Hypertension and Laboratory Markers, Body Composition, Obstructive Sleep Apnea and Autonomic Parameters in Obese Patients

The figures present the data regarding the sensitivity and specificity of systemic arterial hypertension with visceral fat and fasting glucose, respectively, in addition to their cutoff points for screening for systemic arterial hypertension.

## Introduction

Systemic arterial hypertension (SAH) is a multifactorial disease and can be caused by environmental and/or genetic factors, such as lack of physical activity, obesity, and eating habits.^1^According to the World Health Organization, it is estimated that 1.28 billion adults aged 30 to 79 years worldwide have hypertension.^2^Currently, SAH is associated with a higher risk of mortality and is a significant factor in complications of kidney and cardiovascular events.^3^

Hypertension can be induced by possible changes caused by obesity, such as the stimulation of mechanisms that contribute to the hypertensive state, such as hormonal changes and inflammatory and endothelial levels.^4^Obesity is associated with a decrease in life expectancy and its prevalence has become a major worldwide health problem, as excessive weight gain predisposes an increased risk of various diseases, including cardiovascular, cerebrovascular, and metabolic diseases – all of them are associated with SAH.^5 , 6^

Prior literature data describes that some factors should be considered as risk predictors for the occurrence of SAH; among them is obstructive sleep apnea syndrome, body mass index (BMI), waist circumference (WC), VFA, HRV,^7 - 9^some laboratory biochemical markers and associated comorbidities.^10 , 11^Due to the number of variables and their possible associations, further research on relationships between these data and SAH is necessary to obtain more reliable and independent predictors for decision-making in clinical practice, facilitating the prognostic of SAH in this population.

Sleep-related breathing disorders such as obstructive apnea can accelerate the elevation of blood pressure in adults, especially acutely, and may be due to hypoxia at night.^7^The mechanisms caused by a higher value of BMI, WC, body fat, and blood glucose can cause sympathetic nervous system stimulation, changes in the renin-angiotensin-aldosterone system, an increase of inflammatory markers, and other factors responsible for balance in the circulatory system, thus being able to associate with SAH.^12 - 15^Hypertension is also related to autonomic deregulation, and since HRV can also be characterized by greater sympathetic activation, this would be the mechanism associated with SAH.^16^

Given the above, the objective of the present study was to investigate the SAH associations with laboratory biochemical markers, anthropometric and body composition measures, heart rate variability, and obstructive sleep apnea in obese adults, and secondarily, to analyze the sensitivity and specificity of the variables that are independent factors in the association, as well as their respective cut-off points.

## Methods

### Study design and sample

The present study was based on cross-sectional data of 95 patients aged ≥ 21 years with obesity diagnosis and elective to bariatric surgery in a private clinic of surgery and treatment of obesity in the city of Salvador in Brazil. Data were collected from May 2016 to August 2018. This study did not include patients with a cognitive deficit and without all clinical and laboratory data. The study volunteers were categorized into two groups according to the clinical diagnosis of SAH: Normotensive Group (NG) and Hypertensive Group (HG). The study was submitted and approved by the Research Ethics Committee of the Bahiana School of Medicine and Public Health under number 1.530.178. The authors declare that all experiments were conducted following the Declaration of Helsinki.

### Measuring Instruments

#### Body composition

Body composition data were measured by octopolar electrical bioimpedance through the Inbody 720 equipment (Inbody Canada Corp, Ottawa, Ontario, Canada), fulfilling the procedures specified in the literature. The bioimpedance uses eight electrodes: two are positioned in contact with the palm (E1, E3) and the thumb (E2, E4) of each hand, and two are in contact with the anterior (E5, E7) and posterior (E6, E8) of the plant of each foot. Five segmental impedances (right arm, left arm, right leg, left leg, and trunk) are measured at 1, 5, 50, 250, 500, and 1000 kHz. The body contact points were previously cleaned with an electrolytic fabric recommended by the manufacturer, and participants were told to comply with the following preparation standards: to fast for at least 4 hours, no alcohol consumption within 48 hours before testing, no moderate-to-high intensity exercise within 12 hours before evaluation, must be adequately hydrated to perform the exam, must not have metal parts or dental implants (when possible to remove) and no coffee ingestion. As a result of bioimpedance, the following variables were determined: total body mass (kg), body fat mass (kg), skeletal muscle mass (kg), VFA (cm^2^), and body mass index (kg/m^2^). Age (years), height (cm), waist circumference (cm), and hip circumference (cm) were collected from the base of the clinic system medical records.

#### Laboratory biochemical variables

The collected biochemical markers were HOMA-IR, insulin, fasting blood glucose, total cholesterol, HDL cholesterol, and triglycerides. The coloring system quantified total cholesterol, HDL, and triglycerides in the serum. The values of the methodology applied by the laboratory were considered references based on the values presented by the Brazilian Diabetes Society and the *Sociedade Brasileira de Cardiologia.* ^17^All data were collected from the clinic system of medical records preoperatively.

#### Analysis of heart rate variability

A heart rate monitor (V800 Polar Heart Rate Monitor®) was used for cardiac beats, they were calculated through the ratio between the RR interval and transferred to a computer program to analyze HRV through the Polar Precision Performance, which was imported into the Kubios HRV software (version 2.0), and it was used to calculate linear time and frequency domain methods. For the analysis of HRV in the time domain, the square root of the average of square differences between the normal RR intervals (RMSSD) and the standard deviation of the average of all normal RR intervals (SDNN) was used. For HRV analysis in the frequency domain, low-frequency spectral components (LF, 0.04-015 Hz) and high frequency (HF, 0.15 to 0.40 Hz) were used in normal units (LFun and HFun, respectively), which represents a value for each spectral component to the total power minus the very low-frequency components (VLF), and the relationship between these components (LF/HF ratio).

Spectral analysis was calculated using the Fast Fourier Transform algorithm. The sample participants were invited to remain at rest, supine position, without exposure to excessive light, and in a no-noise environment for 10 minutes to analyze a 5-minute cutting point, checking in the preoperative period.

#### Obstructive sleep apnea

Polysomnography data were obtained through computed equipment from Respironics (Healthdyne Alice System 4), and the report was reviewed independently by trained experts. A third expert would be consulted in case of inconsistencies in the final report. The exam was conducted all night, in spontaneous sleep, without sedation or sleep deprivation. It was recorded: electroencephalogram (electrodes C3, C4), oculogram (O1, O2), electromyogram (electrodes in the Mentonian, Submention, and MMII), electrocardiogram, airflow (nasal and oral thermistor), respiratory effort (thoracic and abdominal strap), snoring (microphone on the chin) and body position (sensor in the thoracic strap).^18^

Oxyhemoglobin saturation was measured through pulse oximetry. Respiratory events were thus defined: apnea, such as airflow interruption for 10 seconds or more, and hypopneas, such as the 50% or more reduction of inspiratory airflow per period ≥ 10 seconds, associated with a decrease than 3% in oxyhemoglobin saturation and/or a micro awakening.

Mixed apneas were included in the AHI and defined as those without respiratory effort at the beginning of the period, followed by a gradual increase. The AHI was obtained through polysomnographic examination, dividing the total respiratory events by sleep hours. Patients were classified according to AHI: without apnea - less than 5.0 events/sleep hour; with light apnea - between 5.0 and 14.9 events/sleep time; with moderate apnea - between 15.0 and 30.0 events per/hour of sleep and severe apnea - over 30.0 events/sleep time.

#### Statistical plan

Descriptive and analytical analyzes were performed through the Statistical Package for Social Sciences Program software, Version 14.0 for Windows (SPSS Inc, Chicago, IL). Comparisons between Normotensive patients with SAH were conducted based on clinical diagnosis. The normality of the variables was verified through descriptive statistics and the Kolmogorov-Smirnov test. The categorical variables were expressed in absolute values and percentages, and the chi-square test was used to test the differences between the categorical variables. Continuous variables with normal distribution were expressed as mean and standard deviation, and non-normal distribution as median and interquartile range. Test-T for independent samples or the Mann-Whitney U test was used to test the differences between the continuous variables. Multivariate logistic regression models were used to estimate the association between SAH, body composition, and laboratory markers. The variables that presented p <0.2 were considered for elaborating the adjustment models. The odds ratio has been adjusted to age, sex, height, and oxyhemoglobin saturation. Receiver Operating Characteristic Curve (ROC Curve) were used to estimate the sensitivity and specificity between systemic arterial hypertension, abdominal visceral fat area, fasting plasma glucose, and their respective cutting points. For statistical inference, a value of p<0.05 was adopted.

## Results

A total of 95 participants of both sexes were selected for the study. NG included 57 participants (60%), and HG included 38 participants (40%), with an average age of 36.3 ± 10.1 and 40.4 ± 10.6 years, respectively (p = 0.062). Table 1 presents the characteristics of patients according to the clinical diagnosis of SAH, categorized as NG and HG. Compared to NG, HG had higher body mass, BMI, WC, BFM, and VFA. The percentage of patients diagnosed with insulin resistance was higher in the HG. The groups were homogeneous regarding laboratory data, polysomnography, and the severity of OSAS and HRV parameters.

**Table 1:** – Characteristics of patients according to the diagnosis of systemic arterial hypertension

	NG (n = 57)	HG (n = 38)	p-value
Age (years)	36.3 (10.1)	40.4 (10.6)	0.062
**Gender n (%)**			
Male	15 (26.3)	16 (42.1)	0.123
Women	42 (73.7)	22 (57.9)	
**Body composition**			
Body mass (kg)	113.3 (18.5)	124.6 (25)	**0.013**
Height (cm)	166.8 (8.3)	169.6 (8.4)	0.114
BMI (kg/m^2^)	40.5 (4.6)	42.9 (6)	**0.027**
WC (cm)	117.3 (12.1)	124.6 (18.3)	**0.024**
SMM (kg)	32.7 (7.4)	35.8 (7.6)	0.058
BFM (kg)	55.1 (9.2)	60.7 (14.9)	**0.027**
VFA (cm2)	202.8 (54.1)	262.2 (78.6)	**0.0001**
**Laboratory Data**			
Total cholesterol (mg/dl)	197 (57)	201 (36.7)	0.657
HDL (mg/dl)	49.5 (11.8)	48.3 (11.5)	0.636
Triglycerides (mg/dl)	146 (74)	175.7 (113.4)	0.162
Fasting blood glucose (mg/dl)	96.9 (35.4)	105.5 (26.2)	0.180
Homa-IR	5 (3.9)	6.4 (3.9)	0.135
**Comorbidities n (%)**			
Diabetes Mellitus	5 (8.8)	6 (15.8)	0.338
Insulin Resistance	30 (57.7)	28 (82.4)	0.020
OSAS	34 (65.4)	24 (72.7)	0.633
**Polysomnography measures**			
AHI (events/h)	8.4 [3.8 – 15.7]	9.9 [4 – 17.5]	0.389
OSAS frequency	19 [13.7 – 27.5]	18 [12 – 29.2]	0.754
OS (%)	95 [93 – 96]	94 [92 – 95.7]	0.174
**OSAS severity n (%)**			
< 5 events/h	18 (34.6)	9 (27.3)	0.513
5 – 30 events/h	31 (59.6)	20 60.6)	
> 30 events/h	3 (5.8)	4 (12.1)	
**HRV Parameters**			
**Time-domain**			
Average RR	760.4 [638–849]	726 [647–814]	0.660
SDNN (ms)	70.7 [37.7–274]	84 [43.7–422]	0.522
RMSSD (ms)	64 [21.6–340]	117 [33.8–550]	0.373
pNN50 (ms)	18.4 [1.8–50]	13.6 [3.4–40.4]	0.861
**Frequency domain**			
LF (ms^2^)	56 [41–75]	51 [27–84.8]	0.601
HF (ms^2^)	43.6 [23.6–56]	46 [15.2–64.8]	0.443
LF/HF	1.3 [0.74–3.8]	1.4 [0.54–8.5]	0.898

BMI: body mass index; WC: waist circumference; SMM: skeletal muscle mass; BFM: body fat mass; VFA: visceral fat area; HDL: high-density lipoprotein; AHI: apnea-hypopnea index; OS: oxyhemoglobin saturation; SDNN: standard deviation of the average of all normal RR intervals; RMSSD: square root of the average of square differences between the normal RR intervals; LF: low-frequency; HF: high frequency. Chi-square test was used to analyze diabetes mellitus, insulin resistance, and OSAS; Values presented as mean (standard deviation) or median [interquartile range].

Table 2 presents significant associations (p<0.05) between SAH and measures of body composition, laboratory data, and comorbidities through unadjusted and adjusted multivariate analyzes. The variables body mass, SMM, BMI, WC, triglycerides, HOMA-IR, and insulin resistance showed no statistical differences in multivariate logistic regression analysis. In the final analysis model, after covariable adjustments, including age, gender, stature, and oxyhemoglobin saturation, the association between SAH and body composition was (OR = 0.98, 95% confidence interval (CI) = 0.97-0.99) for the area of visceral fat and laboratory markers (or = 0.96, 95% CI = 0.92-0.99) for fasting plasma glucose. Both variables proved to be the only ones independently associated with HAS.

**Table 2:** – Multivariate logistic regression model of body fat, laboratories, and comorbidity variables among obese patients with and without SAH

Variables	Initial Model			Final Model*		
	
	β	OR (IC 95%)	p	β	OR (IC 95%)	p
Body mass	-0.089	0.91 (0.75 – 1.10)	0.362	-	-	-
Visceral Fat Area	-0.015	0.98 (0.97 – 0.99)	**0.011**	-0.014	0.98 (0.97 – 0.99)	0.026
SMM	0.154	1.16 (0.84 – 1.61)	0.356	-	-	-
BMI	-0.026	0.97 (0.76 – 1.25)	0.842	-	-	-
WC	0.005	1.00 (0.94 – 1.06)	0.882	-	-	-
Triglycerides	-0.001	0.99 (0.99 – 1.00)	0.684	-	-	-
Fasting blood glucose	-0.035	0.96 (0.93 – 0.99)	**0.043**	-0.040	0.96 (0.92 – 0.99)	0.047
Homa - IR	0.017	1.07 (0.86 – 1.19)	0.836	-	-	-
Insulin Resistance	-1.167	0.31 (0.08 – 1.21)	0.093	-	-	-

*The final model includes age, sex, height, and oxyhemoglobin saturation.

Figures 1 and 2 present the data related to the sensitivity and specificity of SAH with the visceral fat and fasting plasma glucose, respectively, in addition to its cutting points for screening of SAH. The visceral fat area had an area under the curve = 0.728 (95% CI = 0.62-0.84) and Cut-off Point for SAH: > 220.3 cm^2^, while fasting plasma glucose presented an area under the curve = 0.69 (95% CI = 0.58-0.80) and the cut-off point for SAH: > 95 mg/dl.

**Figure 1:** – ROC curve for the visceral fat area as screening for SAH. Area under the curve = 0.728; IC 95% (0.620 – 0.836). Visceral fat area cut-off point for SAH: > 220.3 cm2.

**Figure 2:** – ROC curve for fasting blood glucose screening for SAH. Area under the curve = 0.693; CI 95% (0.582 – 0.804). Fasting blood glucose cut-off point for SAH: > 95 mg/dl.

## Discussion

In the present study, comparative analyzes between the groups showed that body composition measures, laboratory data, and comorbidity were higher in the HG. However, the only variables independently associated with SAH were visceral fat area and fasting plasma glucose. The strength of these associations described in the unadjusted analyzes was slightly changed after adjustments to potential confusion variables. These results provide additional support for the importance of maintaining low abdominal visceral fat storage levels, as well as the control of fasting plasma glucose as potential SAH protection factors.

Body composition measures, biochemical markers, and comorbidities evaluated in this study impact the mechanisms related to SAH differently. Pre-studies corroborate our findings. In this sense, Chandra et al.^19^demonstrated that higher measures of BMI were significantly associated with SAH in participants.^19^Still, in this sense, Lee et al.^20^found that at each 1kg/m^2^increase in BMI, there was also a 19% increase in the risk of arterial hypertension, and Holmes et al.^21^showed that for each increase in 1kg/m^2^in IMC the systolic blood pressure increased by 0.70mmHg.^21^A likely explanation for the association of IMC measure with SAH, knowing that it is an index with cutting points for the obesity classification with good accuracy in prediction,^22^is the fact that the obese phenotype, even when metabolically healthy, is directly linked to an increased risk for hypertension,^23 , 24^since there are pathological mechanisms such as hyperinsulinemia, stimulation of sympathetic nervous system and abnormal levels of adipocytokines that affect vascular endothelium, responsible for maintaining vascular homeostasis.^12^

Still dealing with measures of body composition, in our findings, the waist circumference also demonstrated an association with SAH, as well as in the study by Guilherme et al.,^25^which demonstrated that in Brazilian adolescents, the WC obtained a positive association as an independent anthropometric indicator for SAH, and those classified with central obesity were 130% more likely to have high blood pressure compared to adolescents without the diagnosis of abdominal obesity. Carba et al.^26^found that for every 1cm increase in WC, the chances of hypertension increased by 5% for non-overweight women and 3% for overweight women.^26^Since WC is an indicator of abdominal obesity,^27^it can be said that a possible explanation for the association between WC and HAS is related to the excess fat deposits in this part of the body since visceral adipose tissue plays an important role in activating the renin-angiotensin-aldosterone system, which can influence central and systemic hemodynamics.^13^

As we can see, changing a normotensive phenotype to hypertensive involves multiple factors. In addition to the variables already mentioned, BFM also interferes with hemodynamics, so that fat distribution can dictate the risk of cardiovascular disease.^28^Similarly, Han et al.^29^found that the body fat percentage was significantly higher in the hypertensive group 29 compared to normotensive individuals.^29^Park et al.^30^also demonstrated that individuals with a high percentage of body fat were associated with an increased risk of hypertension even with low BMI, WC, or waist-hip ratio, and the increased risk was proportional to the increased percentage.^30^In this case, by increasing BFM, levels in the plasma of inflammatory biomarkers such as C-reactive protein and interleukins may also increase, which may predispose to cardiovascular disease development, including hypertension.^14^

As mentioned earlier, fat distribution can dictate the risk of cardiovascular disease, and, in this sense, individuals with higher levels of visceral adipose tissue and ectopic fat deposits have an even greater prevalence of metabolic disorders such as hypertension.^31 , 32^ Figures 1 and 2 are visualized the area under the ROC curve for sensitivity and specificity for the visceral fat area and fasting plasma glucose found in our study, demonstrating that both variables obtained independent associations with SAH, highlighting mainly the area of visceral fat. Excessive visceral adipose tissue produces hormones and molecules that accentuate cardiovascular disease, become resistant to insulin and leptin, and may contribute to vascular resistance and sympathetic system dysfunction.^31^Intra-abdominal adipose tissue at high levels can be considered as part of a phenotype whose result is associated with a dysfunctional alteration of subcutaneous adipose tissue and ectopic storage of triglyceride, leading to this morphological change to be part of a set of cardiometabolic risk factors.^33^

Once insulin resistance can contribute to vascular resistance and sympathetic system dysfunction,^31^it is important to highlight its relationship with fasting plasma glucose levels since the insulin sensitivity rate decreases as fasting blood glucose increases.^34^In a study conducted in Japan, it was observed that high fasting glucose levels were independently and significantly associated with hypertension, and the risk rate in participants with glucose levels above or equal to 7.0 mmol/L was 1.79 compared to participants with glycemia rate above 5.6 mmol/l.^15^These results corroborate our findings, as fasting plasma glucose has been independently associated with SAH.

The associations between fasting plasma glycemia plus abdominal visceral fat and SAH described in the present study have potential implications for treatment interventions to improve results in obese patients and are probably generalizable for populations worldwide. However, there are limitations to determining whether statistical associations are causal, and the direction of associations should be considered before definitive conclusions are reached. Since the study is observational, it is not possible to rule out the effects of residual or unsuccessful confusion as an explanation for the results. In addition, the transverse design does not allow us to determine whether the clinical picture of SAH preceded or was influenced by the metabolic and morphological profile. The observed SAH associations with biochemical and body markers may be bidirectional.

## Conclusion

In conclusion, the present study demonstrates an inverse and independent association between fasting plasma glucose concentration, abdominal visceral fat area, and SAH in obese patients. In addition, fasting plasma glucose and visceral fat area showed a high sensitivity for SAH screening. The results draw attention to the importance of interventions to improve the control of biochemical and body composition variables, prevent changes in plasma blood glucose, and attenuate increased abdominal visceral fat in obese patients.
